# Alcohol, burn injury, and the intestine

**DOI:** 10.4103/0974-2700.43187

**Published:** 2008

**Authors:** Mashkoor A Choudhry, Irshad H Chaudry

**Affiliations:** 1 Department of Surgery, Burn and Shock Trauma Institute, Loyola University Chicago Medical Center, Maywood, IL 60153, USA; 2 Department of Surgery, Center for Surgical Research, University of Alabama at Birmingham, Birmingham, AL 35294, USA

**Keywords:** Inflammation, tissue damage, trauma

## Abstract

A significant number of burn and other traumatic injuries are reported to occur under the influence of alcohol (EtOH) intoxication. Despite this overwhelming association between EtOH intoxication and injury, relatively little attention has been paid to determining the role of EtOH in post-injury pathogenesis. This article reviews studies which have evaluated the impact of EtOH on post-burn intestinal immunity and barrier functions. The findings from these studies suggest that while a smaller burn injury by itself may not have an adverse effect on host defense, when combined with prior EtOH intoxication it may become detrimental. Experimental data from our laboratory further supports the notion that EtOH intoxication before burn injury suppresses intestinal immune defense, impairs gut barrier functions, and increases bacterial growth. This results in increased bacterial translocation which may contribute to post injury pathogenesis. Altogether, the studies reviewed in this article suggest that EtOH intoxication at the time of injury is a risk factor, and therefore blood EtOH should be checked in burn/trauma patients at the time of hospital admission.

Trauma remains the leading cause of death among people between 1–44 years of age. Burn injury, which is one type of major trauma, is very common at all ages. Nearly one million burn injuries are reported every year in the United States.[1] Burns and trauma are also a major cause of morbidity and mortality in other parts of the world. However, the extent of the problem is poorly documented, particularly in the developing and underdeveloped countries, including in India. Studies have shown that many of these injuries are sustained under the influence of alcohol (EtOH). An analysis of the data indicates that in the United States, nearly half of the burn patients are found positive for blood EtOH at the time of hospital admission.[2–14] A more or less similar proportion of other trauma are also reported to occur in patients under the influence of EtOH.[2,3,11,12,15,16] A few studies have examined the effect of EtOH intoxication on the management of trauma patients. Findings from these studies indicated that intoxicated patients require frequent intubations. Furthermore, they, experience delayed wound healing, and longer hospital stay.[2–16] These findings further suggest that intoxicated patients are more susceptible to infection, and the overall mortality is also relatively higher in intoxicated patients compared to burn patients who have not consumed EtOH prior to injury.[2,3,5–7,9–13] Since the focus of this article is on the intestine, we attempted to review the findings on the effect of EtOH on post-burn intestinal immune and epithelial barrier function.

## EtOH INTOXICATION AND POST-BURN HOST DEFENSE

**Figure 1:**
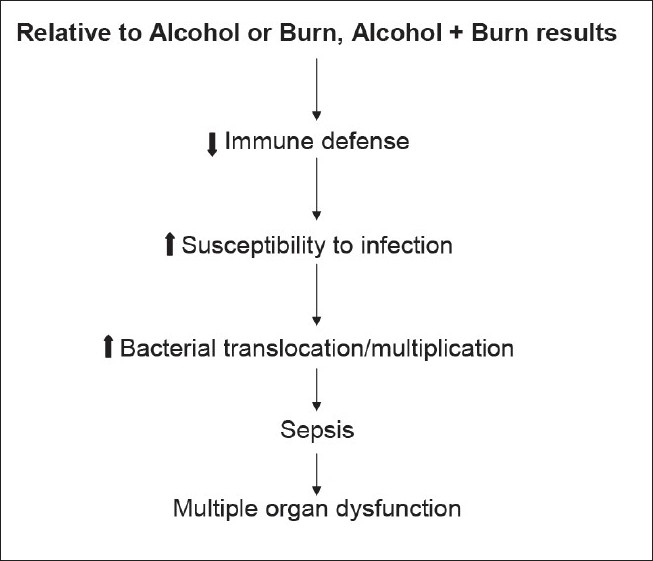
Acute alcohol intoxication exacerbates the suppression in immunity and host defense leading to organ dysfunction that is seen following burn injury

Both experimental and clinical findings indicate that EtOH intoxication has adverse effects on many organs[2,3,11,17–19]; however, the effect of EtOH abuse on morbidity and mortality following burn injury and other trauma remains largely unknown. Studies have shown that in the absence of EtOH intoxication burn injury induces an inflammatory response characterized by uncontrolled production of inflammatory mediators, including cytokines and chemokines, and leukocyte infiltration. However, the adaptive arm of the immune defense, such as antigen presentation, T-cell proliferation, and IL-2 production, is severely suppressed.[20–22] This results in a decrease in host resistance and increase in susceptibility to infection. Similar alterations in immune responses are also reported following EtOH intoxication.[2,11,17–19] In recent years, experimental models were developed to study the impact of EtOH on host defense and other organ functions following burn injury. Results from these studies, as summarized in Figure 1, suggest that acute EtOH intoxication prior to burn injury exacerbates the suppression of host defense and enhances susceptibility to bacterial infection.[2,3,11,17–19] Although, the initial release of cytokines or other inflammatory mediators is a normal host response to injury, if it remains unchecked it can lead to multiorgan dysfunction and failure, which is a major cause of death in injured patients. A common factor in many of these complications is suggested to be the bacteria or their products originating from the intestine. The intestine has therefore become the focus of many studies in the recent past.[23–26]

## EtOH AND POST-BURN INTESTINAL IMMUNITY

The intestine maintains a mucosal and immunological barrier that is normally effective in keeping bacteria within the intestinal lumen. However, these defense mechanisms are impaired in individuals with a history of EtOH abuse and in some disease conditions. The intestinal immune system, also known as the gut-associated lymphoid tissue (GALT), consists of Peyer's patches (PP), mesenteric lymph nodes (MLN), and a large number of immune cells distributed throughout the lamina propria (LP) and epithelium of the intestine.[2] Only few studies could be found in the literature that have examined the effects of EtOH on intestinal immune defense.[2,27,28] The findings from these studies suggested that EtOH consumption is associated with increased intestinal immune cell apoptosis and suppression of immune cell effector response.[2,27,28] Our laboratory has been studying the mechanism by which EtOH intoxication combined with burn injury impairs intestinal immunity. We used a rat model of acute EtOH intoxication and burn injury.[2,29,30] Rats in this model received a single dose of EtOH by gavage feeding of 5 ml of 20% EtOH 4 h prior to burn injury. The results obtained using this model suggest that on day 1 after injury, the suppression in MLN T-cell proliferation and IL-2 production was evident only in the group that had the combined insult of EtOH intoxication and burn injury, while no significant suppression in MLN T-cells was observed in rats receiving either EtOH intoxication or burn injury alone. In contrast, on day 2 after injury, there was a decrease in MLN T-cell function following burn injury alone in the absence of EtOH intoxication, but the suppression was greater in the group receiving combined insult of EtOH intoxication and burn injury than in the rats receiving burn injury alone.[2,29,31–34] These findings collectively suggest that a single dose of acute EtOH may not be sufficient to suppress the T-cell responses; however, in combination with burn injury, EtOH can exacerbate the suppression of T-cell response following the injury. Another study[35] examined the effect of chronic EtOH on post-burn complications; it also compared the effects of enteral (oral) and intravenous administration of EtOH. Rats received 20% EtOH daily for 14 days by gavage (oral) or superior vena cava (intravenous) infusion. Four hours after the final dose of EtOH on day 14, the rats underwent a 30% total body surface area (TBSA) full-thickness burn injury. On day 4 after the burn injury, the rats were killed and various immune and gastrointestinal function parameters were determined. The findings suggest that oral, but not intravenous, administration of EtOH induced significant immunologic and gastrointestinal dysfunction. Furthermore, chronic EtOH abuse with or without burn injury results in a significantly greater suppression of lymphocyte proliferation, damage to intestinal mucosa, and increase in bacterial translocation.[35] These results suggest that both acute and chronic EtOH ingestion prior to burn injury may result in a synergistic alteration of intestinal immunity and barrier function.

## EtOH AND POST-BURN INTESTINAL BARRIER FUNCTION

EtOH is known to cause toxic effects in the liver, pancreas, and gastrointestinal tract.[36–41] Absorption of EtOH occurs throughout the entire intestinal mucosa, but studies indicate that absorption is faster in the duodenum and jejunum.[36,41–44] There is evidence that both acute and chronic EtOH consumption affects the structure and function of the entire gastrointestinal tract.[36,41–43] Acute administration of EtOH results in the loss of epithelium at the tips of the villi, hemorrhagic erosions, hemorrhage in the lamina propria, and inflammatory cell infiltration.[45] However, the degree of mucosal damage is dependent on the dose of EtOH and the manner in which EtOH is administered.

Similarly, chronic EtOH ingestion has been found to cause damage in the mucosa of the small intestine. Studies have shown that rats gavaged daily with 3 ml of 20% EtOH for 4 and 8 weeks exhibited flattening and blunting of villi in the jejunal mucosa.[41] These changes in intestinal morphology were accompanied with an increase in intestinal permeability. In an another study, Keshavarzian *et al*.[46] showed that chronic EtOH consumption reversibly affects the integrity of small intestinal villi in men. In yet another study, Rossi *et al*.[47] have shown that alcoholic animals exhibited conspicuous alterations in their mitochondria and endoplasmic reticulum. Altogether, these findings suggest that EtOH abuse impairs the intestinal barrier function.

The intestine is continuously exposed to the outside environment. It is a major reservoir of nutrients, proteolytic enzymes, bacteria, and bacterial toxins. Under healthy conditions, the intestine maintains a mucosal barrier, which permits selective absorption of nutrients into the circulation. This barrier also functions as a local defense, preventing bacteria and endotoxin contained within the intestinal lumen from translocating to extraintestinal sites. However, this mucosal barrier is impaired in various pathologic conditions, including in burns and trauma.[48–54] Increased bacterial translocation is the most common marker of intestine tissue injury.

Many studies have indicated that burn injury causes gut mucosal atrophy, alters mucosal integrity, and deteriorates intestinal mucosal barrier function.[2,50,55–59] It was found that a small burn injury (< 20% TBSA) did not induce bacterial translocation.[53] In contrast, a relatively larger burn area (40% TBSA) caused a significant increase in viable bacterial translocation. Similarly, Fazal *et al*.[50] have indicated that there is enhanced translocation of viable bacteria into the mucosal tissue, PP, and MLN at day 1 after injury in rats receiving 30% TBSA burn injury. Thus, the severity of the injury may be a factor in post-burn pathogenesis.

Only few studies in the literature have examined the effect of EtOH intoxication on the intestine barrier following burn injury. In one study,[35] rats were fed on EtOH by gavage for 14 days prior to receiving a 30% TBSA burn injury. The translocation of bacteria was determined 4 days after burn injury. The results show a significant increase in bacterial translocation in rats receiving a combined insult of EtOH intoxication and burn injury compared with rats receiving either EtOH intoxication or burn injury alone. Studies in our laboratory have evaluated the effect of a single dose of EtOH (5 ml of 20% EtOH) on post-burn intestinal barrier function.[29,31,33,60] We found that as compared to control, rats receiving a single dose of EtOH exhibited an increase in intestinal permeability on day 1 but not on day 2.[29,31,33] In contrast, burn injury (12.5% TBSA) alone did not influence the intestine permeability on day 1 after injury. However, a tendency for an increase in intestinal permeability was observed in rats receiving burn injury alone on day 2 after injury as compared to sham controls. The combined insult of EtOH and burn injury resulted in a significant increase in intestinal permeability on both days.[29,31,33,60] This was accompanied by an increase in the number of bacteria in the MLN. Thus, it appears that EtOH intoxication before injury acts synergistically with the injury to adversely affect intestinal barrier functions and thus increase bacterial translocation.

## EtOH AND POST-BURN INTESTINAL BACTERIAL GROWTH

Under healthy conditions, a few indigenous bacteria continuously translocate to the MLN, but because of the intact immune defense these bacteria do not survive. Thus, the MLN from normal animals remains relatively sterile. However, EtOH ingestion, with or without burn injury, disrupts the effective mucosal defense, leading to the passage of viable bacteria across the luminal barrier to the MLN and distant organs. The definitive pathways by which bacteria reach the MLN and systemic organs following EtOH and burn injury are uncertain and remain to be established. ‘M cells’ which are the specialized epithelial cells in the PP may serve as a carrier for translocating bacteria.[61] The pathogens, once they cross the gut epithelial barrier through M cells, come in contact with the immune cells of the PP and are cleared if the immune cells are functioning normally. However, when PP immune cell functions are not intact, these bacteria are not cleared; rather, the immune cells (e.g., macrophages or dendritic cells) may become the carrier and transport the bacteria from the PP to the MLN and from the MLN to the systemic circulation. As reviewed in a previous article,[2] studies have shown spontaneous gut bacterial translocation to the MLN, spleen, and liver in athymic (nu/nu) mice, whereas no translocation was noticed in heterozygous (nu/+) or nude (+/+) mice grafted with thymus. To determine whether T-cell-dependent immunity is critical for defense against enteric bacteria, we performed an experiment using rats in which T-cells were depleted prior to EtOH intoxication and burn injury. The data obtained from this experiment suggest that the depletion of CD3+ cells in healthy rats caused an increase in the bacterial accumulation in the MLN. Furthermore, CD3+ cells depletion in EtOH plus burn-injured rats appeared to promote the spread of bacteria to the spleen and the systemic circulation.[29]

## EtOH AND POST-BURN INTESTINAL BACTERIAL GROWTH

**Figure 2:**
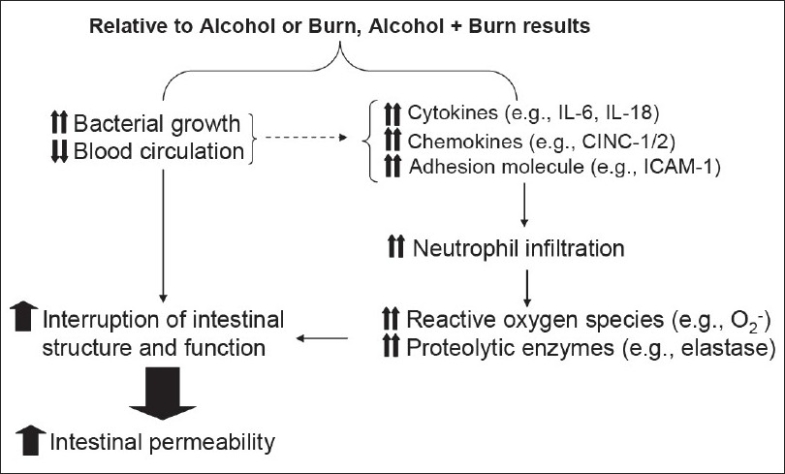
Diagrammatic representation of the events leading to increased intestine permeability following a combined insult of alcohol and burn injury

Under physiological conditions, nearly 10^12^-10^15^ bacteria are present in the intestine.[62,63] Of these nearly 10^9^are potentially pathogenic gram-negative enteric bacteria. Under healthy conditions, these bacteria normally reside in the lower part of the gastrointestinal tract. Studies have shown that individuals with a history of heavy EtOH use had an increase in intestinal bacterial growth, and in many cases these bacteria started colonizing the upper part of the gastrointestinal tract.[2,64,65] In one study, 89 patients with alcoholic cirrhosis and 40 healthy subjects were enrolled to assess the prevalence of intestinal bacterial overgrowth and its relationship with the severity of liver dysfunction.[66] The findings from this study suggested intestinal bacterial overgrowth in nearly 30% (27 out of 89) of the patients with alcoholic cirrhosis. Furthermore, the study concluded that intestinal bacterial overgrowth occurs in patients with advanced liver dysfunction. Although the mechanism by which EtOH influences bacterial growth remains unclear, there are reports suggesting that neurochemicals can directly influence the growth and virulence of bacteria.[67] Since both EtOH intoxication and burn injury influence hormonal levels, it is likely that these changes in hormone levels may affect bacterial growth. However, studies are needed to confirm such a role for hormones in the increase in bacterial growth or virulence. Another possibility is that individuals with a history of EtOH abuse have impaired intestinal peristalsis, which increases the likelihood of bacterial growth. In one study we examined the effect of acute EtOH intoxication on post-burn intestinal bacterial growth [Figure 2]. The finding from this study indicates that although administration of a single dose of EtOH did not influence the intestine bacterial number, it significantly enhanced the bacterial growth following burn injury.[31]

The question whether increased bacterial growth causes increase in bacterial translocation following EtOH, either alone or in combination with burn injury, remains to be answered. A few studies have suggested that anaerobic intestinal bacteria are 100- to 1000-fold more numerous than the gram-negative enteric bacteria and are closely associated with the intestinal epithelial lining.[62,63] The presence of this large number of anaerobic bacteria prevents gram-negative enteric bacteria from gaining attachment to the intestinal epithelial lining. Furthermore, studies have also shown that this ratio of anaerobic bacteria to gram-negative bacteria changes following ingestion of broad-spectrum antibiotics, to which the anaerobes are more sensitive.[62,63] Such a decrease in anaerobic bacterial numbers increases the possibility of the attachment of gram-negative pathogens to intestinal epithelial lining and their subsequent translocation to extra-intestinal sites. Although it is not clear whether EtOH influences this ratio, findings from the studies suggest an increase in gram-negative bacteria following EtOH abuse.[64,68] Thus, any change in intestinal bacterial growth, as is observed following EtOH intoxication plus burn injury, may result in bacterial translocation [Figure 2]. However, more studies are needed to confirm this paradigm.

## EtOH AND POST-BURN INTESTINE INFLAMMATORY RESPONSE

Several lines of evidence indicate that EtOH intoxication, as well as injury such as burn and trauma, results in an increase in the inflammatory mediators. As summarized in Figure 2 these include, for example, the infiltration of leukocytes, such as neutrophils; the production of cytokines, such as IL-6 and IL-10; and chemokines IL-8, MIP, MCP-1 cytokine-induced neutrophil chemoattractant (CINC), and adhesion molecules (e.g., ICAM). Furthermore, the increase in these inflammatory mediators has been proposed to contribute to tissue damage under those conditions.[50,56,57,68–80] Previous findings from our laboratory have shown that acute EtOH intoxication combined with burn injury enhances IL-18 production in various lymphoid organs and in the intestine.[33,81] There was also an increase in intestinal tissue neutrophil infiltration, neutrophil chemokines, edema, and permeability.[33,60] Moreover, we found that animals treated with anti-IL-18 antibody did not exhibit an increase in intestine neutrophil infiltration, tissue edema, or increase in intestinal permeability following a combined insult of EtOH and burn injury. Similarly, other cytokines/chemokines such as IL-6, MIP, and MCP1 may also help in neutrophil recruitment. The depletion of neutrophils prior to injury prevented the increase in intestinal MPO activity, edema, and permeability after EtOH and burn injury but it did not influence IL-18 levels.[33,81] Similarly, neutrophil presence was found to be critical for the increase in intestinal permeability following burn injury.[58,59] Thus, the increase in cytokines or chemokines following major injury helps in the recruitment of neutrophils, which in turn may play a predominant role in tissue injury following major trauma and burn injury, as well as following EtOH intoxication. Neutrophil-mediated oxidant injury is demonstrated in other pathologic conditions, such as rheumatoid arthritis, acute respiratory distress syndrome, and tissue ischemia. Although an exact mechanism by which neutrophils may cause tissue damage following major injury remains to be established, studies have shown that under normal conditions neutrophils release oxygen radical species and proteolytic enzymes to kill pathogens. In various inflammatory conditions, such as burn, trauma, and acute and chronic EtOH ingestion, neutrophils are hyperactive, resulting in the excessive release of oxygen radical species and proteolytic enzymes.[33,58,82–85] Such excessive release of neutrophil reactive oxygen species and proteases has been implicated in organ tissue damage [Figure 2].

## EtOH AND POST-BURN INTESTINE BLOOD FLOW

In addition to increased inflammatory mediators, changes in blood circulation to the intestine may produce alterations in intestine immune and epithelial barrier functions following EtOH and burn injury [Figure 2]. Such changes in blood circulation to the intestine could result from redistribution of the blood supply to some organs versus others. Many studies have shown that following major trauma or injury, blood supply to some organs is markedly reduced in order to maintain the functioning of the vital organs. Studies have shown that blood flow to the intestinal bed is significantly decreased following major burn injury.[52,86–90] There are two phases of cardiovascular response to burn injury.[71,72] The initial phase, referred to as the ‘hypovolemic phase,’ is characterized by decreased blood flow to tissues and organs. This is followed by a hyper-metabolic phase that is characterized by increased blood flow to the tissues and organs. Studies have also indicated that the hemodynamic response to burn injury is dependent on the size of the burn area.[86,87] Carter *et al*.[73] did not observe differences in intestinal and hepatic blood flow after 20% TBSA burn injury. Similarly, the studies of Ferguson *et al*.[76] did not show significant differences in liver and intestinal blood flow following burn injury. We examined the effect of EtOH intoxication on post-burn intestinal blood flow.[91] We did not observe a significant effect of 12.5% TBSA burn injury on hemodynamic responses. In contrast, hemodynamic responses were significantly altered when the 12.5% TBSA burn injury was combined with prior EtOH intoxication. Our findings suggest that the cardiac output and blood flow in the liver and small intestine was significantly decreased in rats receiving a combined insult of EtOH and burn injury compared to rats receiving either sham or burn injury alone.

The oxygen delivery to the liver and small intestine was also significantly decreased following EtOH plus burn injury compared to either EtOH or burn injury alone. The oxygen extraction and consumption, on the other hand, was significantly enhanced in both the organs. This difference in oxygen delivery and oxygen consumption may cause hypoxic insult to the liver and intestine. Thus, following EtOH and burn injury, such ischemic conditions may create an inflammatory environment which, in turn, may cause tissue damage in the intestine and other organs.[74,79,84–86]

## CONCLUSIONS AND FUTURE RECOMMENDATIONS

The studies reviewed in this article indicate a significant association between EtOH intoxication and traumatic injuries, including burns. However, a clear picture of the role of EtOH in post-injury complications remains to be established. Findings obtained using experimental models of EtOH and burn injury indicate that a small burn injury may not produce significant alterations in immune and other organ functions; however, when it combines with prior EtOH intoxication, it becomes detrimental. Studies have also indicated that acute EtOH intoxication prior to burn injury exacerbates the suppression of intestinal immunity and deteriorates gut permeability following burn injury. There was a significant decrease in the intestine blood flow following a combined insult of EtOH intoxication and burn injury. A significant increase in bacterial growth was also observed in the intestine of rats receiving a combined insult of EtOH intoxication and burn injury. Such alterations in intestinal immunity and barrier functions may cause an increase in bacterial translocation, which in turn may contribute to organ dysfunction and pathogenesis in the injured host. Thus, studies are needed to delineate the mechanism by which alcohol intoxication combined with burn injury produces alterations not only in intestinal immunity and barrier functions but other organs also. Studies are also needed to determine whether age and gender has any role to play in the pathogenesis following alcohol and burn injury. The results from these studies will help in designing therapeutic approaches specific for the treatment of burn and trauma patients with EtOH history.
